# Presentation, treatment and clinical outcomes in young multiple myeloma patients treated at a tertiary care centre in a low middle-income country

**DOI:** 10.3332/ecancer.2025.1978

**Published:** 2025-08-29

**Authors:** Tasneem Dawood, Sahar Fatima Rizvi, Syed Muhammad Kashif Kazmi, Saqib Raza Khan, Insia Ali, Munira Moosajee

**Affiliations:** 1 Division of Medical Oncology, Department of Oncology, Aga Khan University Hospital, Karachi 74800, Pakistan; 2 Department of Nephrology, National Institute of Kidney and Urological Disease, Karachi 75270, Pakistan; 3 Division of Medical Oncology, Department of Oncology, Schulich School of Medicine & Dentistry, Western University, London, ON N6A3K7, Canada; 4 Verspeeten Family Cancer Centre, London Health Sciences Centre, London, ON N6A5W9, Canada; a https://orcid.org/0000-0002-3742-534X

**Keywords:** multiple myeloma, young patients, clinical characteristics, treatment patterns, survival outcomes

## Abstract

**Background:**

Multiple myeloma (MM), a plasma cell malignancy, predominantly affects individuals aged 65–74 years. However, its occurrence in younger populations (<55 years) is rare, posing unique challenges. This study explores the clinical presentation, outcomes and treatment regimens for MM patients aged 30–55, aiming to unravel age-specific patterns.

**Methods:**

A retrospective chart review was conducted at Aga University Hospital, Karachi, focusing on MM patients aged 30–55 years. Data included patient demographics, clinical features, treatment modalities and outcomes. Statistical analysis employed STATA version 16.0, incorporating survival estimates, log-rank tests and Cox proportional survival regression models.

**Results:**

This study encompassed 68 confirmed MM patients, categorised by age groups: 30–40 years (13.23%), 41–50 years (44.11%) and 51–55 years (42.64%). Predominantly male (1.3:1 ratio), bone pains were prevalent among all groups, with 51–55 years exhibiting the highest pathological fracture rate. 30–40 years group showed higher renal impairment rates and lactate dehydrogenase levels. Velcade, thalidomide and dexamethasone were commonly used first-line regimens in the entire cohort in 41.17% of patients, closely followed by cyclophosphamide, bortezomib and dexamethasone in 29.41%. Partial response was predominant in the 30–40 years group, while other age groups showed varied responses. The younger patients demonstrated lower deep treatment response rates than their older counterparts. Progression-free survival was 37, 52 and 45 months orderly in each group, with a *p*-value of <0.001. The median overall survival (OS) for the entire group was 50.6 months (4.2 years), with OS rates of 77.8% (CI: 95%), 90.0% (CI: 95%) and 86.2% (CI: 95%), respectively, with a *p*-value of <0.001. Median OS in months was 45, 55.5 and 52, with a *p*-value of 0.08.

**Conclusion:**

This single-center study sheds light on younger MM patients' unique challenges and treatment patterns. Despite the rarity of this age group's affliction, the findings underscore significant differences in clinical presentations, treatment responses and outcomes compared to the typical elderly MM population. The study highlights the importance of tailored approaches in managing MM across different age brackets, emphasising the need for further research to optimise therapeutic strategies and improve prognosis in this distinct patient cohort.

## Introduction

Multiple myeloma (MM) is characterised by the proliferation of clonal plasma cells in the bone marrow, and this results in various complications such as bone destruction, renal impairment, anemia and hypercalcemia [1]. MM has been recognised as a disease predominantly affecting the elderly, with the highest incidence reported in individuals aged 65 to 74 years. The risk increases with age, reaching over 40 cases per 100,000 among those over 80. The median age at diagnosis is approximately 69 years. However, MM isn't limited to older adults; although it's rare, younger people can also develop the disease. Around 10% of MM cases are found in individuals under 50 and only 2% in those under 40 [2–4]. This younger group of patients faces unique challenges and needs different treatment strategies than older patients.

The uncommon occurrence of MM in younger patients (under 55 years old) makes it challenging to fully understand its clinical behaviour and devise ideal treatment protocols. Younger MM patients often have different disease characteristics, presentation and response to therapy compared to older patients [5]. For example, younger patients usually have fewer comorbidities, better performance status and may tolerate aggressive treatments better; these differences can impact their clinical outcomes and survival rates. Additionally, young patients face significant impacts on their productive years, including prolonged treatment toxicity and unique challenges related to symptom burden, overall life impact and financial strains [5]. The reduction in life expectancy for these patients can be significant, up to 36 years for those under 40 and 27 years for those aged 40–49 [6, 7].

Despite significant advancements in treatment, MM remains a challenging and mostly incurable disease [8–11]. Current treatment strategies focus on controlling the disease, relieving symptoms, preventing organ damage, improving quality of life and prolonging survival. Over the past few decades, the introduction of novel therapies—such as proteasome inhibitors (e.g., bortezomib and carfilzomib), immunomodulatory drugs (e.g., thalidomide, lenalidomide and pomalidomide) and monoclonal antibodies (e.g., daratumumab and elotuzumab)—has transformed the management of MM, leading to improved survival rates. Standard treatment regimens frequently include a combination of these novel agents with corticosteroids and chemotherapy, often tailored to the patient's condition and disease stage.

Autologous hematopoietic stem cell transplantation (AHSCT) remains a cornerstone of therapy for transplant-eligible MM patients, particularly younger individuals who can better tolerate intensive treatment. Despite improvements in progression-free survival (PFS) and overall survival (OS), relapses remain common due to residual disease. Whether following AHSCT or used in non-transplant settings, maintenance therapy aims to prolong and deepen the responses achieved through initial treatment [12].

Treatment choice often depends on various factors, including disease stage, cytogenetic abnormalities and patient comorbidities. For younger patients, the aim is to achieve the deepest possible response, as achieving a complete response (CR) or stringent complete response (sCR) is associated with prolonged PFS and OS. The goal for younger patients should be to enhance long-term outcomes while minimising treatment-related side effects. However, the best treatment approach for younger MM patients is still an active area of research.

In younger patients, the survival rates tend to be better than older patients, likely due to their ability to undergo aggressive treatments and overall better health status. However, despite these advantages, younger MM patients face a significant disease burden and the potential for long-term complications related to both the disease and its treatment.

Young MM patients are underrepresented in the literature and often overshadowed by older cohorts in clinical trials. Few studies have specifically addressed the clinical characteristics and outcomes of younger MM patients, with most research comprising case reports or small cohorts over several years [5, 13]. The lack of comprehensive studies, with the rapid advancements in therapeutic, diagnostic and biological understanding, underscores the need for a detailed approach to treating this subpopulation. Given this, our study aims to provide valuable insights into disease biology and optimal treatment strategies for this distinct and often overlooked subgroup of MM patients [9, 10].

## Methods

This retrospective study was done at the Aga University Hospital in Karachi. The study population comprised young individuals with symptomatic MM from ages 30–55. For the study analysis, they were grouped into ages 30–40, 41–50 and 51–55 years. Patients in the smoldering/asymptomatic phase of MM and those deemed extremely ill to undergo treatment were excluded.

Retrospective data encompassing 2012–2020 were extracted from the medical records of all patients diagnosed with MM within our institution. A structured pre-formed proforma guided the systematic collection of pertinent variables, ensuring a comprehensive and standardised approach to the dataset. STATA version 16.0 was employed for data analysis, involving descriptive statistics for continuous and categorical variables. Mean and standard deviation summarised continuous variables and frequency counts/percentages reported categorical ones. Kaplan–Meier estimates and the log-rank test assessed survival function, while a Cox proportional survival regression model was used for comprehensive analysis. Initial univariate analysis gauged variable significance (<0.25), followed by multicollinearity checks. Significant variables, free of multicollinearity, underwent multivariable analysis (<0.05), including scrutiny for plausible interactions.

## Results

A total of 68 patients with a confirmed diagnosis of MM were included in this analysis. The patient population was stratified by age group, of which 9 (13.23%) patients were 30–40 years of age (Group 1), 30 (44.11%) patients were 41–50 years of age (Group 2) and 29 (42.64%) were aged 51–55 years (Group 3). The median ages in each age group were 35, 47.5 and 54 years, respectively. There was a male predominance with a male-to-female ratio of 1.3:1 in the entire cohort, with a much higher ratio in group 1 of 8:1. Bone pains (62/68 – 91.17%) were the most common presenting symptom in all three groups. The rate of pathological fracture was the highest in group 3 (12.5% versus 66.6% versus 81.25%, *p* = 0.29), though the difference between groups 2 and 3 was not statistically significant. Group 1 had higher rates of renal impairment than groups 2 and 3 (*p* = <0.01), as well as renal failure (55.6% versus 40% versus 34.5%). Lactate Dehydrogenase (LDH) was also significantly higher in Group 1 than in 2 and 3 (447 versus 307 versus 368 U/L *p* value <0.001). There were no significant differences in gender, hemoglobin levels, calcium levels, heavy or light chain restriction or International Staging System (ISS) stage. Around 40% of patients in all groups were diagnosed with Stage III. IgG myeloma was more common than non-IgG myeloma in each group (Table 1).

Most of the patients underwent treatment with novel agents. Velcade, thalidomide and dexamethasone (VTD) were the most common induction treatment of choice for the entire cohort, received by 41.17% of the patients, followed by cyclophosphamide, bortezomib and dexamethasone (CyBorD) in 29.41% (Table 2). Treatment selection was guided by a combination of factors, including drug availability, cost considerations, institutional protocols and national guidelines. These determinants played a key role in shaping the therapeutic approach.

Regarding the response assessment after induction treatment, as shown in Table 2, a partial response (PR) was demonstrated in 77.8% of the patients and no very good partial response (VGPR), CR or sCR was observed in group 1 and progressive disease (PD) was observed in 22.2%. For group 2, PR, VGPR and CR sCR were observed in 53.3%, 10%, 6.7% and 6.7%, respectively, and PD was observed in 23.3% of the patients. In group 3, PR, VGPR, CR and sCR were observed in 55.2%, 5%, 5% and 3.5%, respectively, and PD in 6.9%. Out of the 68 patients, only 6 (8.8%) underwent transplant. Most (80%) patients received maintenance treatment, as shown in Table 2. The overall response rate post-induction (ORR: CR/VGPR/PR/sCR) was similar in the first two groups, 77.8% and 76.7%, but significantly higher in group 3 (93.1%).

**Figure 1.**
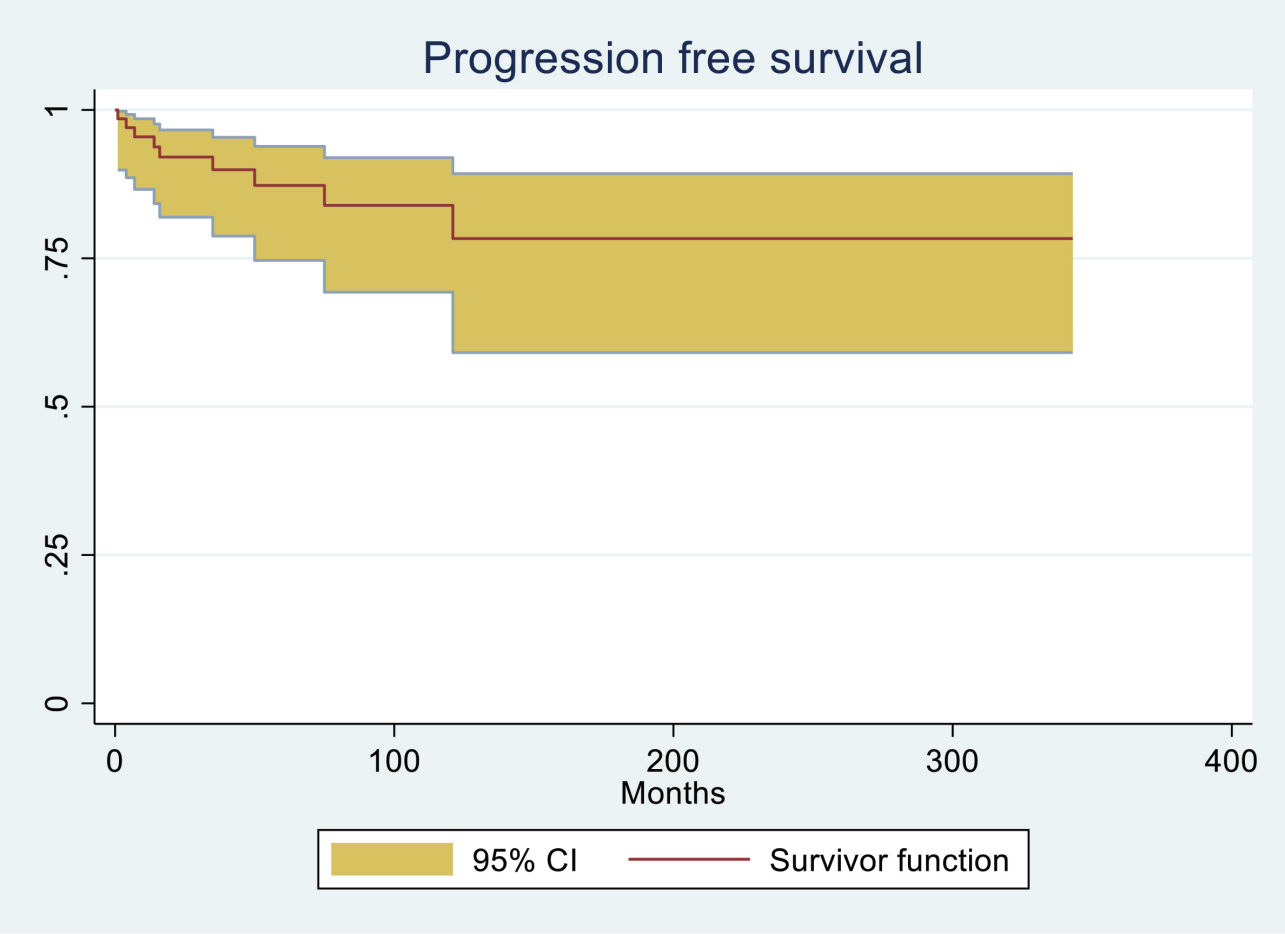
PFS (in months) for the entire cohort of MM patients.

**Figure 2.**
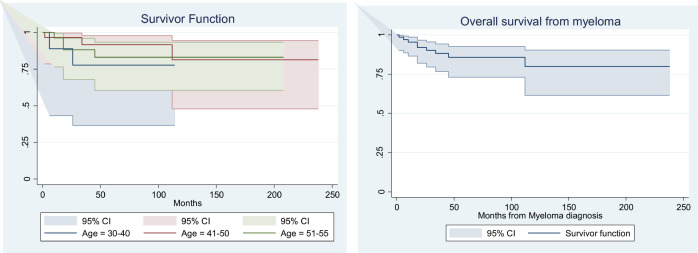
(a): OS (in months) for MM patients in different age groups. (b): OS (in months) for the entire cohort of MM patients.

The median follow-up duration for the entire cohort was 57 months. PFS was significantly longer for groups 2 and 3 compared to group 1, with a median PFS of 37 months for group 1, 52 months for group 2 and 45 months for group 3 (*p* < 0.001), as shown in Table 3. The median PFS for the entire cohort was 44.4 months (*p* = < 0.001) (Figure 1). The median OS in months for each group was 45, 55.5 and 52, respectively (*p* = 0.08) (Table 3, Figure 2a). The median OS for the entire population was 50.6 months (4.2 years) (Figure 2b), with OS rates of 77.8%, 90.0 % and 86.2%, respectively, with a (*p* = <0.001) (Table 4).

## Discussion

The present study investigates the presentation, clinical outcomes and treatment regimens utilised in young patients diagnosed with MM. Since MM is rare in young individuals, a focused approach is required to understand this age group's specific patterns and challenges. The median age of diagnosis for MM is commonly reported to be between 65 and 74 years, making the inclusion of younger patients, aged 30–55 years and a distinctive aspect of this research.

Regarding clinical characteristics and outcomes, this study included 68 patients younger than 55 with a median follow-up of 57 months. Similar studies focusing on young myeloma patients have included comparable participant populations. For instance, a study enrolled 61 patients under 60 years of age, while Yanamandra *et al* [14] had 40 patients under 40 and Jurczyszyn *et al* [15] investigated 52 patients under 30. Some notable findings concerning patient characteristics and disease presentation were observed across different age groups. Renal failure was more prevalent among younger patients (30–40 years) in our study than in groups >40 years. While Veerendra *et al* [16] reported renal failure in 17.2% of younger MM patients and 35.8% of the elderly, their study did not directly compare age groups within the same analytical framework as ours [16]. Additionally, the incidence of pathological fractures was lower in the younger age category in our study. At the same time, Butler *et al* [17] found that pathological sternal fractures are more common in younger myeloma patients with thoracic compression fractures [17]. These observations suggest age-specific variations in disease presentation.

Our analysis of treatment regimens showed that VTD was the most frequently used first-line regimen, followed closely by CyBorD. Various studies, such as those by Leiba *et al* [18] and Moreau *et al* [19] have also highlighted the superiority of VTD therapy in achieving deeper responses. The median OS for the entire cohort in our study is 50.6 months. In comparison, reviews of similar studies suggest that the median OS for MM patients ranges widely from 7 months to over 76 months, depending on factors such as age, treatment regimen and disease stage [13, 19–21]. Distinct OS rates were observed among different age groups in our study. Notably, the 30–40 age group exhibited decreased OS and PFS compared to patients aged 41–55.

In contrast, Jurczyszyn *et al* [22] demonstrated that younger myeloma patients aged 21–40 have better OS than those aged 41–60, although this survival advantage diminishes in more advanced stages. Despite generally favourable prognoses in some malignancies, younger patients in our study exhibited poorer OS, challenging older analyses suggesting better survival rates for those younger than 50 years [23]. This emphasises the complex and multifactorial nature of MM outcomes.

The treatment patterns in this study indicate that a relatively low percentage of patients undergoing transplants (8.8%) raises questions about the comprehensive management of young myeloma patients. In contrast, other studies show significantly higher transplant rates in young MM patients; notably, Caulier *et al* [24] found that 25% of young patients had allogeneic stem cell transplantation, and Aytan *et al* [25] reported 83.9% of young patients undergoing transplants. The low transplant rates in our study may reflect survivorship-related issues such as financial burden, infertility, risks of secondary malignancies and other factors influencing treatment decisions in this population.

The limitations of this study include its retrospective nature and relatively small sample size. The data sparsity made it difficult to conduct thorough univariate and multivariate analyses, indicating a need for larger studies to validate our findings. Additionally, the study did not explore specific genetic or molecular markers, which could provide valuable insights into the heterogeneity of myeloma in young patients.

Despite these limitations, this study sheds light on the unique characteristics of MM in young individuals. The observed age-specific differences in clinical presentation, treatment patterns and outcomes emphasise the importance of developing tailored therapeutic approaches for this population. The findings also point to significant challenges, such as a higher incidence of renal failure and reduced survival rates in the 30–40-year-old age group, which merit further investigation and should be considered when designing personalised treatment strategies for young myeloma patients.

In recent years, the treatment landscape for MM has evolved significantly with the advent of novel therapies that have improved patient outcomes. These include proteasome inhibitors, immunomodulatory drugs, monoclonal antibodies and chimeric antigen receptor T-cell therapy [26, 27]. These advancements offer promising avenues for addressing MM's complex and heterogeneous nature, particularly in younger patients who may benefit from more aggressive and targeted approaches. Ongoing research into the integration of these novel therapies and the exploration of specific genetic and molecular markers holds the potential to further enhance the precision and effectiveness of treatments for young myeloma patients.

## Conclusion

This single-center study sheds light on younger MM patients' unique challenges and treatment patterns. Despite the rarity of this age group's affliction, the findings emphasise significant differences in clinical presentations, treatment responses and outcomes compared to the typical elderly MM population. The study highlights the importance of tailored approaches in managing MM across different age brackets, emphasising the need for further research to optimise therapeutic strategies and improve prognosis in this distinct patient cohort.

## List of abbreviations

CR, Complete response; CRAB criteria, Calcium elevation, renal insufficiency, anemia, bone lesions; CyBorD, cyclophosphamide, bortezomib, dexamethasone; ISS, International Staging System; LDH, Lactate dehydrogenase; LEN/DEX, Lenalidomide/Dexamethasone; MM, Multiple myeloma; MPV, Melphalan, prednisone, velcade; NA, Not applicable; OS, Overall survival; PD, Progressive disease; PFS, Progression-free survival; PR, Partial response; R-ISS, Revised International Staging System; sCR, Stringent complete response; THAL/DEX, Thalidomide/Dexamethasone; VGPR, Very good partial response; VTD, Velcade (Bortezomib), thalidomide, dexamethasone.

## Conflicts of interest

All the authors declare no competing interest.

## Funding

No funding is available.

## Consent for publication

Not applicable.

## Ethics approval and consent to participate

The Aga Khan University Hospital Ethical Review Committee (AKUH-ERC) approved our study. Due to the study's retrospective nature, the Ethical Review Committee of Aga Khan University Hospital, Karachi, Pakistan, waived the need for informed consent.

## Availability of data and materials

The data that has been used is confidential. The datasets are available from the corresponding author.

## Disclosure

Artificial intelligence tools were not used in the writing or preparation of this manuscript. The authors generated all the content.

## Author contributions

TD, SK: Conceptualisation, Writing, Performing the experiment, contributing reagents, analysing and interpreting the data.

SKK: Contributed reagents and analysed the data.

SFR, IA: Perform the experiment, contribute reagents and analyse and interpret the data.

MM: Conceptualisation. Perform the experiment and supervise the manuscript.

TD, MM: Conceived and designed the experiments.

## Figures and Tables

**Table 1.** Patient characteristics associated with MM (*n* = 68).

Variables	MM patients			*p*-value
	Group 1 30–40 years (*n* = 9)	Group 2 41–50 years (*n* = 30)	Group 3 51–55 years (*n* = 29)	
**Age, years** **Median**	35	47.5	54	-
**Sex of the participant** ** Male** **Female**	8 (88.9) 1 (11.1)	16 (53.3) 14 (46.7)	15 (51.7) 14 (48.3)	0.13
**Bone pain** **No** **Yes**	1 (11.1) 8 (88.9)	1 (3.3) 29 (96.7)	3 (10.7) 25 (89.3)	0.39
**Fracture** **No** **Yes**	8 (88.9) 1 (11.1)	18 (60.0) 12 (40.0)	16 (55.2) 13 (44.8)	0.29
**Cord compression** **No** **Yes**	9 (100.0) 0 (0.0)	29 (96.7) 1 (3.3)	28 (96.6) 1 (3.4)	0.59
**Anemia** **No** **Yes**	4 (44.4) 5 (55.6)	13 (43.3) 17 (56.7)	14 (48.3) 15 (51.7)	0.80
**Renal failure** **No** **Yes**	4 (44.4) 5 (55.6)	18 (60.0) 12 (40.0)	19 (64.5) 10 (34.5)	0.97
**Hypercalcemia** **No** **Yes**	7 (77.8) 2 (22.2)	22 (73.3) 8 (26.7)	25 (86.2) 4 (13.8)	0.20
**Skeletal** s**urvey** **No** **Yes**	1 (11.1) 8 (88.9)	0 (0.0) 30 (100.0)	2 (6.9) 27 (93.1)	0.51
**Serum** i**mmunofixation** **IgG** k**appa** **IgG** l**ambda** **IgA** k**appa** **IgA** l**ambda** **Kappa** l**ight** c**hain** **Light** c**hain** l**ambda** l**ight** c**hain** **Non** s**ecretory**	4 (44.5) 3 (33.3) 0 (0.0) 1 (11.1) 1 (11.1) 0 (0.0) 0 (0.0)	9 (30.0) 5 (16.7) 3 (10.0) 4 (13.3) 5 (16.7) 3 (10.0) 1 (3.3)	14 (48.3) 6 (20.7) 1 (3.5) 1 (3.5) 3 (10.2) 2 (6.9) 2 (6.9)	0.35
**Hemoglobin (g/d**L**)** **Median**	9.0	10.1	9.5	-
**Creatinine (mg/d**L**)** **Median**	1.6	1.1	1.0	-
**LDH (u/**L**)** **Median**	447	307	368	-
**Albumin (g/d**L**)** **Median**	3.6	3.5	3.4	-
**ISS- staging** **NA** **Stage I** **Stage II** **Stage III**	1 (11.1) 3 (33.3) 1 (11.1) 4 (44.5)	1 (3.3) 12 (40.0) 5 (16.7) 12 (40.0)	2 (6.9) 8 (27.6) 9 (31.0) 10 (34.5)	0.74

LDH: Lactate Dehydrogenase, ISS: International Staging System

**Table 2.** Treatment pattern and responses in patients with MM (*n* = 68).

Initial treatment given VTD CYBORD RD MPV	Group 1 (30–40 years) 4 (44.4) 4 (44.5) 1 (11.1) 0 (0.0)	Group 2 (41–50 years) 10 (33.3) 12 (40.0) 8 (26.7) 0 (0.0)	Group 3 (51–55 years) 14 (48.3) 4 (13.8) 10 (34.5) 1 (3.4)	*p*-value 0.18
**Number of** c**hemotherapy cycles** **Median**	4	4	4	
**Treatment response after** i**nduction** **PR** **VGPR** **CR** **sCR**	7 (77.8) 0 (0.0) 0 (0.0) 0 (0.0)	16 (53.3) 3 (10.0) 2 (6.7) 2 (6.7)	16 (55.2) 5 (17.2) 5 (17.2) 1 (3.5)	0.987
**PD**	2(22.2)	7(23.3)	2(6.9)	
**Autologous transplant** **No/NA** **Yes**	7 (77.8) 2 (22.2)	27 (90.0) 3 (10.0)	28 (96.5) 1 (3.5)	0.27
**Maintenance therapy given** **Yes** **No/NA**	8 (88.8) 1 (11.1)	22 (73.3) 8 (26.6)	25 (86.2) 4 (13.8)	0.782
**Type of maintenance therapy** **LEN/DEX** **THAL/DEX**	3 (33.3) 3 (33.3)	12 (40.0) 8 (26.6)	13 (44.8) 11 (37.9)	0.985
**LEN**	2(22.2)	1(3.3)	1(3.4)	
**BOR**	0(0)	1(3.3)	0(0)	
**Lines of treatment** **<3** **3**	5 (55.6) 4 (44.4)	18 (60.0) 12 (40.0)	18 (62.1) 11 (37.9)	0.71

Velcade, Thalidomide, Dexamethasone (VTD), CyBorD (Cyclophosphamide, Bortezomib, Dexamethasone) Lenalidomide, Dexamethasone (RD), Melphalan, Prednisone, Velcade (MPV), CR: Complete Response, VGPR: Very Good Partial Response, PR: Partial Response, sCR: Stringent Complete Response PD Progressive Disease

**Table 3.** Combined survival outcomes by age group in MM patients.

	MM patients			*p*-value
	Group 1 30–40 years	Group 2 41–50 years	Group 3 51–55 years	
Median PFS (in months)	37 months	52 months	45 months	<0.001
Median OS (in months)	45 months	55.5 months	52 months	0.08

**Table 4.** Survival rate (%) stratified on age group.

	MM patients			*p*-value
	Group 1 30–40 years	Group 2 41–50 years	Group 3 51–55 years	
Survival rate (%)	77.8	90.0	86.2	<0.001
